# Borate-guided ribose phosphorylation for prebiotic nucleotide synthesis

**DOI:** 10.1038/s41598-022-15753-y

**Published:** 2022-07-19

**Authors:** Yuta Hirakawa, Takeshi Kakegawa, Yoshihiro Furukawa

**Affiliations:** Department of Earth Science, Tohoku University, 6-3, Aza-aoba, Aramaki, Aoba-ku, Sendai, 980-8578 Japan

**Keywords:** Astrobiology, Origin of life, Astrobiology, Geochemistry, Geochemistry

## Abstract

Polymers of ribonucleotides (RNAs) are considered to store genetic information and promote biocatalytic reactions for the proto life on chemical evolution. Abiotic synthesis of ribonucleotide was successful in past experiments; nucleoside synthesis occurred first, followed by phosphorylation. These abiotic syntheses are far from biotic reactions and have difficulties as a prebiotic reaction in reacting chemicals in a specific order and purifying intermediates from other molecules in multi-steps of reactions. Another reaction, ribose phosphorylation followed by nucleobase synthesis or nucleobase addition, is close to the biotic reactions of nucleotide synthesis. However, the synthesis of ribose 5′-phosphate under prebiotically plausible conditions remains unclear. Here, we report a high-yield regioselective one-pot synthesis of ribose 5′-phosphate from an aqueous solution containing ribose, phosphate, urea, and borate by simple thermal evaporation. Of note, phosphorylation of ribose before the nucleoside formation differs from the traditional prebiotic nucleotide syntheses and is also consistent with biological nucleotide synthesis. Phosphorylation occurred to the greatest extent in ribose compared to other aldopentoses, only in the presence of borate. Borate is known to improve the stability of ribose preferentially. Geological evidence suggests the presence of borate-rich settings on the early Earth. Therefore, borate-rich evaporitic environments could have facilitated preferential synthesis of ribonucleotide coupled with enhanced stability of ribose on the early Earth.

## Introduction

Abiotic formation of functional polymers with catalytic capabilities and information-carrying capacities would have been essential for the emergence of primitive life. The discovery of the catalytic RNAs supports that RNA might have served these functions in the primordial life, as proposed by the RNA world hypothesis^1,2^. Abiotic formation of RNA monomers (i.e., ribonucleotides) on the prebiotic Earth is considered as an essential step in the prebiotic RNA synthesis. However, the process of the abiotic formation of ribonucleotides on the Hadean Earth needs further elucidation.

Nucleotides have been chemically synthesized via the formation of nucleosides and subsequent phosphorylation of these molecules^3–12^. In abiotic nucleoside syntheses, condensation between ribose **1** and nucleobase results in the formation of purine nucleosides in low yields and pyrimidine nucleosides in no detectable yields^3^. A high yielding nucleoside synthesis needs step-by-step reactions of small reactive molecules with separation and purification of the intermediate chemical compounds^4–6^; the geological settings/events that would have permitted such step-wise synthesis remain unclear. On the other hand, phosphorylation of nucleosides occurs by simple drying of nucleoside and phosphate **2** with catalysts such as urea **3**^7–12^. Ribose **1** can form via a simple condensation of formaldehyde, which can, in turn, be formed by a photochemical reaction between CO_2_ and H_2_O^13,14^. These hint at a possibility of phosphorylation of ribose **1** before the nucleoside formation on Hadean Earth. Further, phosphorylation of sugar before the nucleoside formation is seen in nucleotide biosynthesis in extant life. Phosphorylation of sugar is also an essential reaction related to metabolism in all living cells. Given this, we investigated the phosphorylation of ribose **1** under prebiotically plausible condition.

The sugar phosphate backbone of RNA consists of phosphate **2** and ribose **1** with phosphodiester linkages at 5′- and 3′-hydroxyl groups on ribose **1**. Simple heating of ribose **1** and phosphate **2** with cyanogen **4** selectively yields ribose 1′-phosphate **5**^15^. Further, the reaction of ribose **1** with amidotriphosphate selectively yields ribose 1′,2′-cyclic phosphate^16^. The synthesis of ribose 1′-phosphate **5** was also demonstrated by artificial electrospray-ionized microdroplets using ribose **1** and phosphate **2**^17^. The synthesis of ribose 5′-phosphate **6** has only been achieved by similar artificial electrospray-ionized microdroplets using ribose **1** and pyrophosphate^18^. Nonetheless, the availability of electrospray-ionized microdroplets on the Hadean Earth is questionable. Thus, the synthesis of biologically relevant ribose-phosphates (i.e., ribose 5′- and 3′-phosphate) under plausible prebiotic conditions remains elusive.

Urea **3**, a simple amide molecule, has been used as an efficient abiotic catalyst for the phosphorylation of nucleosides^8,19^. In addition, previous research reported that urea **3** could be a nucleobase precursor on the prebiotic Earth^20,21^. Urea **3** is formed via hydrolysis of cyanamide that can form from ammonia and cyanide^19^. Both ammonia and cyanide can be formed by impact-induced reactions^22^. These reactions would have been common on the Hadean Earth when the impacts of large meteorites and asteroids were more frequent than today^22,23^. Thus, it is reasonable to assume urea as a prebiotically available molecule on the early Earth. However, the effects of urea **3** on the phosphorylation of sugars have not been investigated. Therefore, we investigated the phosphorylation reaction of aldopentoses in the presence of urea **3**.

Sugars are readily decomposed by heating. In particular, ribose is the least stable aldopentose^24^. Previous studies have found that several oxyanions can improve the stability of sugars^13,25,26^. In particular, borate **7** can strongly stabilize ribose **1** compared to other aldopentoses^27,28^. This indicates that borate-rich environments could have been advantageous for nucleotide formation as ribose **1** could have accumulated in such environments. Furthermore, borate **7** is known to control the phosphorylation site of nucleosides^29^. The effects of borate **7** on other steps involved in prebiotically plausible nucleotide synthesis have been reported in many previous studies^6,30,31^. Therefore, we investigated the effects of borate **7** on the phosphorylation of ribose **1** and other aldopentoses under evaporation.

## Results

**Figure 1:**
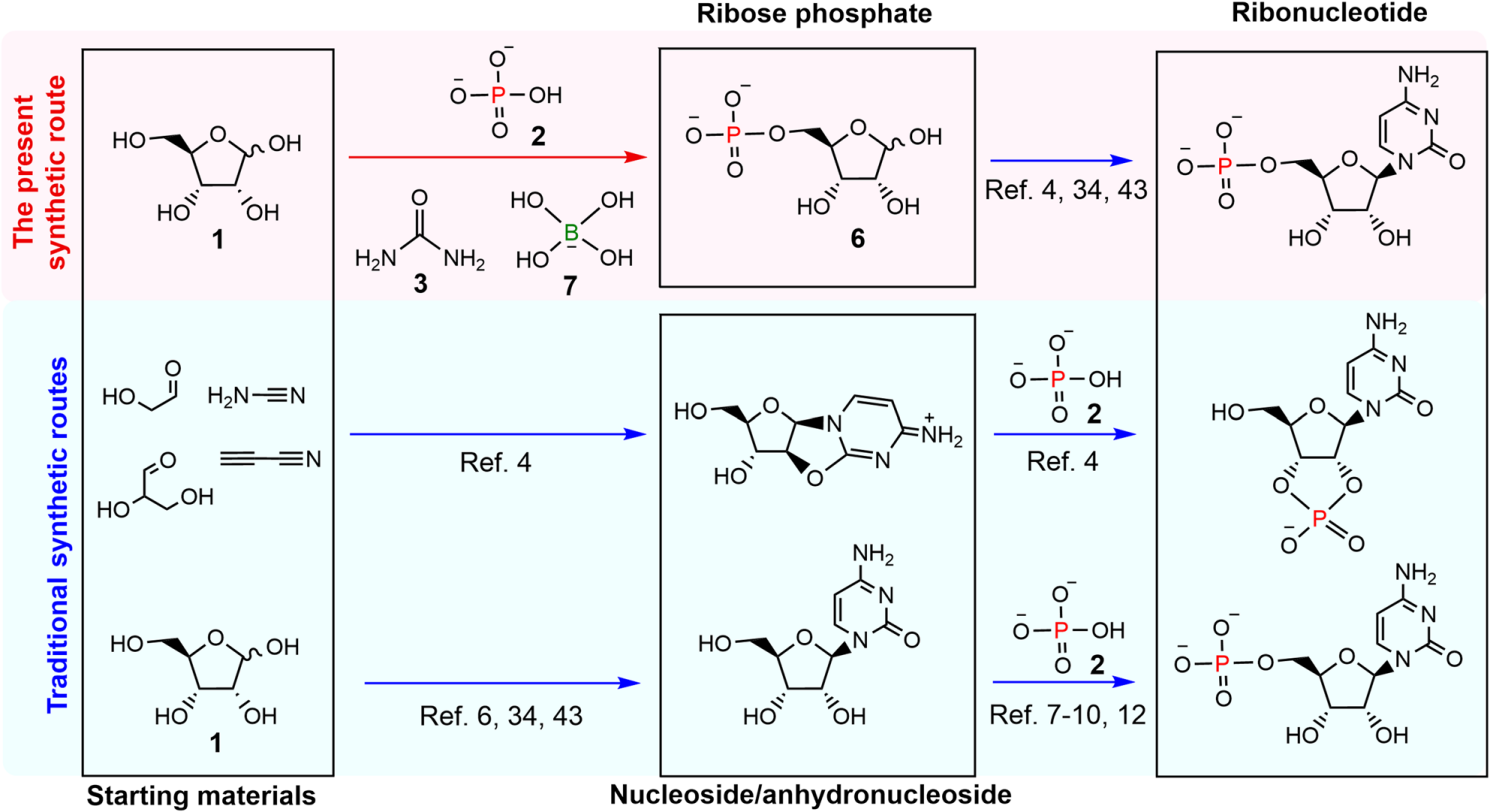
The present and traditional synthetic routes for abiotic nucleotide formation. Red and blue arrows represent the reactions in this study and previous studies, respectively.

**Figure 2:**
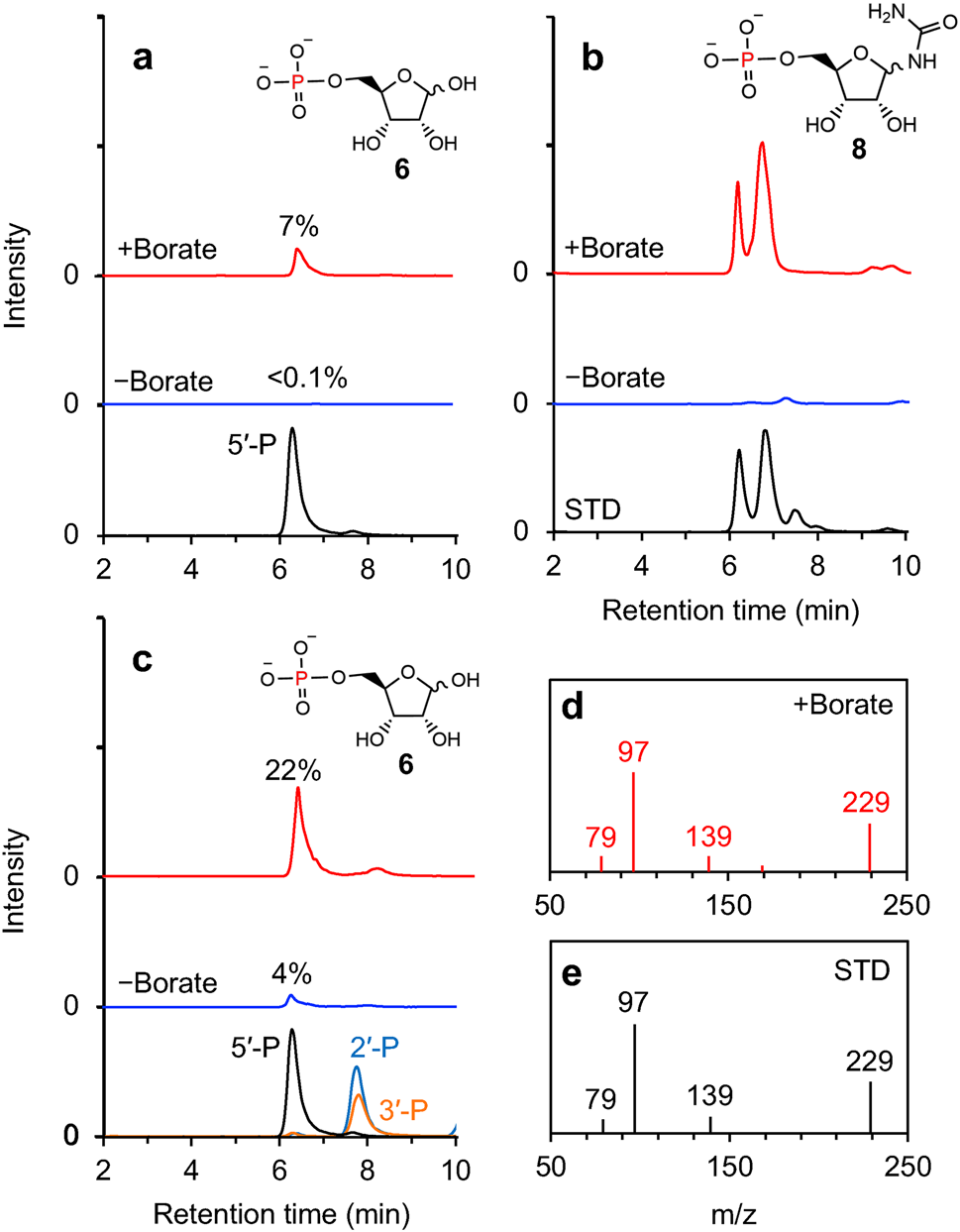
Identification of ribose-phosphate and phosphorylated ribosylurea by LC–MS/MS. (**a**) Ribose-phosphate in acidified product solution (*m/z*: 229.1). (**b**) Ribosylurea phosphate in acidified product solution (*m/z*: 271.1). (**c**) Ribose-phosphate after acid hydrolysis (*m/z*: 229.1). (**d**) Fragment mass spectrum of ribose 5′-phosphate formed in the reaction containing boric acid. The *m/z* of 229 and 97 are ribose-phosphate and phosphate, respectively. (**e**) Fragment mass spectrum of standard ribose 5′-phosphate (STD).

Here, we show the regioselective synthesis of ribose 5′-phosphate **6** from ribose **1** and dissolved phosphate **2** in the presence of urea **3** and borate **7** under simple drying conditions (Fig. 1). A neutral aqueous solution containing d-ribose, disodium monophosphate, urea, and boric acid (pH 8) was dried by heating at 80 °C for 24 h. The precipitates were hydrolyzed in acidic water (pH ~ 1) at 90 °C for 1 h and analyzed using high-performance liquid chromatography-tandem mass spectrometry (LC–MS/MS). The formation of significant amount of ribose 5′-phosphate **6** (i.e., 22 mol% yield on average; n = 3; 1σ =  ± 2.5) in the reactions containing boric acid was confirmed by its LC retention time, MS/MS fragmentation pattern, and ^31^P-NMR (Fig. 2c–e, Figs. S1, S2, and S3). In the reaction with no boric acid, a small amount of ribose 5′-phosphate **6** (i.e., 4 mol% yields) was detected (Fig. 2c). A small amount of ribose 5′-phosphate **6** (i.e., 7 mol% yields) was also detected in the product formed with boric acid before acid hydrolysis. Any ribose phosphate was not formed in the acid condition from ribose and phosphate (Fig. S4). The yield of ribose 5′-phosphate **6** in the reaction without boric acid before acid hydrolysis was < 0.1 mol% (Fig. 2a). In the mass chromatogram corresponding to the molecular mass of ribosylurea phosphate **8**, two significant peaks, indicating the presence of ribosylurea phosphate **8**, were detected only in the reaction containing boric acid before acid hydrolysis (Figs. 2b and S5). Amines tend to react with aldehydes to form imines. Thus, we envisage that 1′-aldehyde of ribose **1** reacted with urea **3** to form ribosylurea **9**^32^.

## Discussion

**Figure 3:**
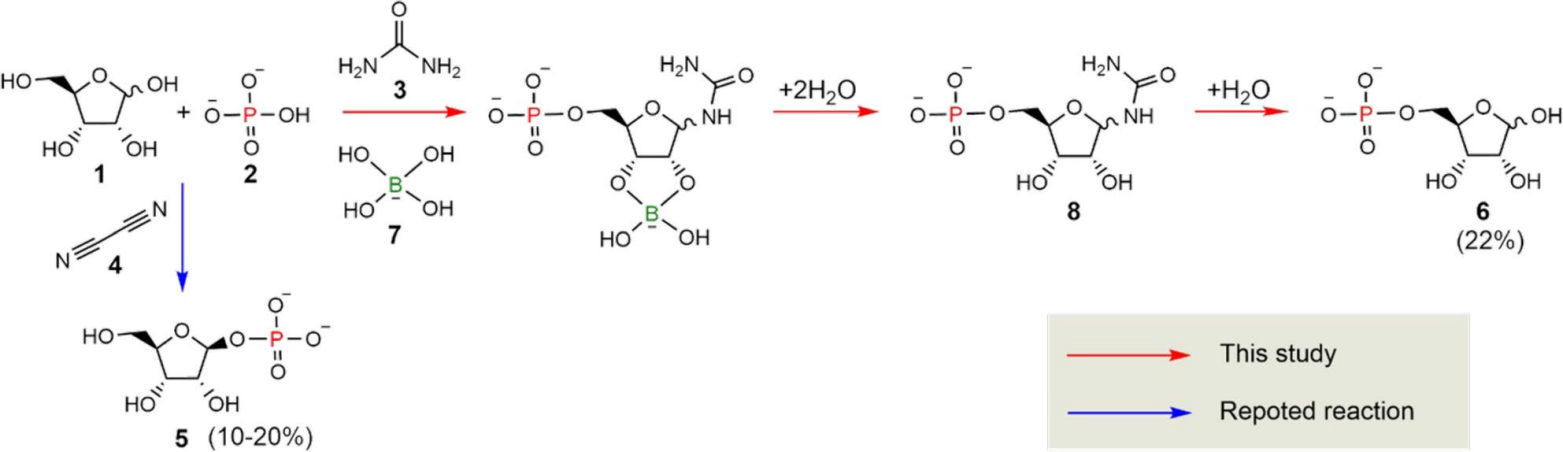
Borate-guided one-pot regioselective phosphorylation of ribose at 5′-hydroxyl position.

These results indicate that ribose **1** was regioselectively phosphorylated at the 5′-hydroxyl group in the presence of borate **7**. The residual fraction of ribose **1** was found to be 19 mol% in the presence of borate **7** and 3 mol% in the absence of borate **7**. This shows that borate improved the stability of ribose **1** in the reaction (Fig. S6). Borate **7** forms a complex with a diol facing the same direction. In the case of ribose **1**, complex formation with its 1′- and 2′-hydroxyl groups improves the stability of ribose **1** by fixing its form in furanose structure^33^. In the present reaction, urea **3** reacted with 1′-hydroxyl of ribose **1**^32^. Thus, borate **7** might have formed a complex with ribose **1** at its 2′, 3′-diol. The complex formation fixed ribose **1** in the furanose form and improved its stability. This led to the high yielding regioselective phosphorylation of ribose **1** at its 5′-hydroxyl (Fig. 3). Phosphorylation at 5′-hydroxyl limits the form of ribose exclusively in furanose, whereas the form of ribose in 2′-phosphate, 3′-phosphate, and 2′,3′-cyclic phosphate can be both furanose and pyranose.

This one-pot synthesis of ribose 5′-phosphate **6** is a simpler reaction than multistep nucleoside syntheses and indicates that ribose phosphorylation could have occurred before the nucleoside formation on the Hadean Earth, in particular, in borate-rich environments. A previous study showed the formation of α-cytidine nucleotide from ribose 5′-phosphate using cyanamide and cyanoacetylene^34^. Another previous study reported that a photochemical reaction can convert the α-nucleotide to canonical β-nucleotide^4^. Thus, nucleotide formation from ribose 5′-phosphate is possible using small reactive molecules via a photochemical reaction, although the net yield is unclear^4,34^. Thus, the ribose phosphate synthesis shown in this study opens a new route for prebiotic ribonucleotide synthesis. This route is geochemically more plausible than traditional prebiotic nucleotide syntheses and consistent with extant biological nucleotide synthesis.

**Figure 4:**
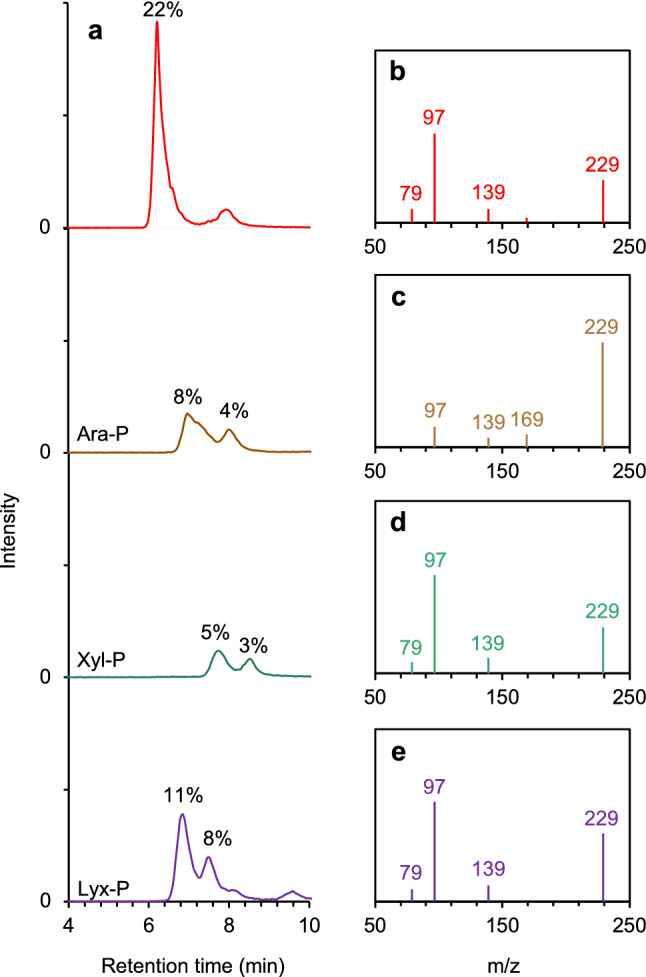
Selective phosphorylation of ribose in borate containing reaction mixture. (**a**) Mass chromatograms and yields of pentose-phosphates in experiments containing borate (*m/z*: 229.1). (**b**) Fragment mass spectrum for ribose 5′-phosphate. (**c**) Fragment mass spectrum for arabinose-phosphates. (**d**) Fragment mass spectrum for xylose-phosphates. (**e**) Fragment mass spectrum for lyxose-phosphates.

We further evaluated the phosphorylation of other aldopentoses (i.e., arabinose **10**, xylose **11**, and lyxose **12**; Fig. S7) in the presence of borate **7**. The phosphorylated products of aldopentoses other than ribose **1** showed multiple peaks in LC–MS/MS chromatograms, indicating that the phosphorylation occurred at different hydroxyls on each aldopentose (Fig. 4). The extent of phosphorylation was evaluated based on the area under the peak of phosphorylated aldopentoses, assuming that the ionization efficiency of these compounds is similar to that of ribose 5′-phosphate **6** (Fig. S8). Ribose **1** was selectively phosphorylated at 5′-hydroxyl group with the highest yield of 22 mol%, whereas other pentoses were phosphorylated at varied hydroxyl positions with total yields of 11, 8, and 19 mol% for phosphorylated arabinose **10**, xylose **11**, and lyxose **12**, respectively (Fig. 4). Selectivity of phosphorylation was not apparent in the reactions carried out without borate **7** (Fig. S9). Therefore, the borate-guided phosphorylation of pentoses shows the preferential synthesis of ribose 5′-phosphate **6**.

The residual fraction of each aldopentose was 19, 41, 22, and 16 mol% for ribose **1**, arabinose **10**, xylose **11**, and lyxose **12**, respectively, in the presence of borate **7** (Fig S7). Compared to other aldopentoses, the rate of phosphorylation based on the residual amounts of pentoses and their phosphorylated products was found to be higher for ribose **1** and lyxose **12** (Figs. 4, S7, S9, and S10). Ribosylurea **9** and lyxosylurea **13** tend to react with borate **7** at their 2′, 3′-diol and get fixed in their respective furanose forms^33^ because borate **7** forms a more stable complex with diols than with single hydroxyl groups^33^. The remaining 5′-hydroxyl group can then react with phosphate **2** (Fig. S11). On the other hand, arabinosylurea **14** and xylosylurea **15** might remain in their chain forms when these molecules are combined with borate **7** due to different directions of hydroxyls on these molecules, as compared to those on ribose **1** and lyxose **12** (Fig. S11). Because the chain form of ureido-pentoses can also combine with borate **7** at their 3′, 4′-diol and 4′, 5′-diol positions in addition to 2′, 3′-diol position, the phosphorylation can get partially inhibited by these borate-diol complexes. These differences in the forms of complexes with borate **7** might underly the different phosphorylation efficiencies observed for varied pentoses as well as the highest phosphorylation observed for ribose **1** (Fig. S11).

Tourmaline, a boron-rich mineral, has been found in > 3.7-billion-year-old metasediments in Isua, Greenland^35,36^. Further, the likely presence of early evaporitic environments has been reported^37^. These early Archean geological conditions presumably extended back to the Hadean Earth. Borate **7** has been thought to have been present in the Hadean oceans as well; this might have led to borate **7** accumulation in evaporitic environments on the Hadean Earth^38^. Ribose **1** might have preferentially accumulated over other aldopentoses in such environments as borate **7** is known to contribute to the stabilization of ribose **1**^13,38^. A higher concentration of carbonate than present is expected in the Hadean ocean, covered by a CO_2_-rich atmosphere. In such an ocean, higher phosphate **2** concentrations than today are expected due to the consumption of Ca^2+^, a cation to form Ca^2+^-phosphate, as Ca^2+^-carbonate^39–42^. The evaporation processes could have also accumulated dissolved phosphate **2** and urea **3** in the same place as borate **7** and ribose **1** and further induced dehydration reaction leading to the phosphorylation of ribose **1**. The present results indicate that the borate-rich evaporitic environment on prebiotic Earth could have enabled the preferential synthesis of ribose 5′-phosphate **6** before the formation of nucleosides.

Previous research reported nucleotide formation from ribose 5′-phosphate **6** using small reactive molecules or amino acids^35,43^, although the nucleotide synthesis through ribose 5′-phosphate **6** needs further investigation. Furthermore, many previous papers reported nucleoside formation from ribose under prebiotically plausible conditions^3,6,30,44,45^. The literature indicates that nucleotide formation using ribose 5′-phosphate **6** could be possible on the prebiotic Earth. Therefore, the one-pot synthesis of ribose 5′-phosphate **6** opens the renounced abiotic route of ribonucleotide formation. This route is more geochemically plausible and analogous to its biosynthesis (Fig. 1). This may provide a geochemical explanation regarding how ribose **1**, the least stable aldopentose^24^, became the selected sugar in RNA.

## Methods

### Materials

Reagent grade d-ribose, urea, boric acid, sulfuric acid, sodium hydroxide, disodium phosphate, and dipotassium phosphate were obtained from Wako Pure Chemical Industries (Osaka, Japan). Regent grade d-ribose 5′-phosphate disodium salt dihydrate, adenosine 2′-monophosphate, and adenosine 3′-monophosphate were obtained from Sigma-Aldrich and used as standards. All chemicals were used without further purification. LC–MS grade ammonium formate (Sigma-Aldrich) and acetonitrile (Merck) were used for LC–MS/MS analyses. Water was purified using Milli-Q Integral (18.2 MΩ·cm and < 3 ppb TOC).

### Preparative synthesis of ribose-phosphate and ribosylurea

LC–MS/MS standards for ribose 2′-phosphate and ribose 3′-phosphate were prepared by heating 20 mM of 2′-AMP and 3′-AMP, respectively, at 90 °C for 1 h in 2 wt% sulfuric acid solution to hydrolyze N-glycosidic bond between ribose and urea. LC–MS/MS standard of ribosylurea phosphate was prepared by heating 20 mM of ribose 5′-phosphate and 80 mM urea at 60 °C for 24 h.

## Experiments

The phosphorylation experiments were conducted in Eppendorf tubes. The 20 µL reaction mixture contained 20 mM ribose, 800 mM urea, 40 mM boric acid, and 160 mM disodium phosphate in water. The starting materials were heated at 80 °C for 24 h with the lids of the tubes kept open. After heating, the residues were resuspended in 200 µL of water. Borate was removed from the product by adding 4 µL sulfuric acid (95%) to the resuspended sample solution before LC–MS/MS analysis. For investigating the acid hydrolyzed product, a sulfuric acid solution containing the experimental residue was heated at 90 °C for 1 h with the lid closed. The hydrolysis would have progressed slowly in the prebiotic ocean, where pH was close to neutral, and the temperature was lower than the acid treatment, although the rate of reaction should be lower.

The control experiment in acidic conditions was conducted in Eppendorf tubes. 20 µL of reaction mixture containing 20 mM ribose, 800 mM urea, 40 mM boric acid, and 160 mM disodium phosphate in acidic water adjusted pH ~ 1 with sulfuric acid was dried down at 80 °C. The experimental and analytical procedures were the same as other experiments. For investigating the effect of acid treatment on phosphorylation, the starting material before the experiment was diluted to tenfold with pure water, adjusted at pH ~ 1 with sulfuric acid, and heated at 90˚C for 1 h.

### HPLC analysis

The separation and detection of the products were conducted using Shimadzu LCMS 8040 (Kyoto; Japan) with the hydrophilic interaction chromatography mode using HILICpak VT-50 2D column (5 μm, 2.0 mm ID, 150 mm length; Shodex). The sample was eluted with isocratic elution with an aqueous solution containing 80% 25 mM ammonium formate and 20% acetonitrile at a total flow rate of 0.2 mL/min at 60 °C. Mass spectrometry was conducted in negative mode with desolvation, source, and heat block temperatures set at 250 °C, 120 °C, and 400 °C, respectively.

### ^31^P-NMR analysis

^31^P-NMR spectra were acquired with Bruker AVANCE III 500 spectrometer. High concentration samples necessary for the NMR analysis were obtained by the following method. The 20 µL aqueous solution containing 200 mM ribose, 4 M urea, 400 mM boric acid, and 1.6 M dipotassium phosphate was heated at 80 °C for 24 h with the lids of the tube kept open. Dipotassium phosphate was used instead of disodium phosphate due to its better solubility. After heating, the residues were resuspended in 200 µL of water, added by 4 µL sulfuric acid (95%), and heated at 90 °C for 1 h. Before the ^31^P-NMR analysis, pH was adjusted to about 8 with sodium hydroxide solution.

## Supplementary Information

Supplementary Figures.

## Data Availability

All data are available in the main text and supplementary information.
