# Supplementary material for: Borate-guided ribose phosphorylation for prebiotic nucleotide synthesis
Source: Sci Rep. 2022 Jul 19;12:11828. doi: 10.1038/s41598-022-15753-y (PMC9296462; doi:10.1038/s41598-022-15753-y)
Supplement: Supplementary file 1 — Supplementary Figures. [file 41598_2022_15753_MOESM1_ESM.pdf]

Supplementary Information for
Borate-guided ribose phosphorylation for prebiotic nucleotide synthesis

Yuta Hirakawa, Takeshi Kakegawa, Yoshihiro Furukawa

Yuta Hirakawa, Yoshihiro Furukawa
Email: yuta.hirakawa.s2@dc.tohoku.ac.jp; furukawa@tohoku.ac.jp

This PDF file includes:

Figures S1 to S11

Supplementary Figures

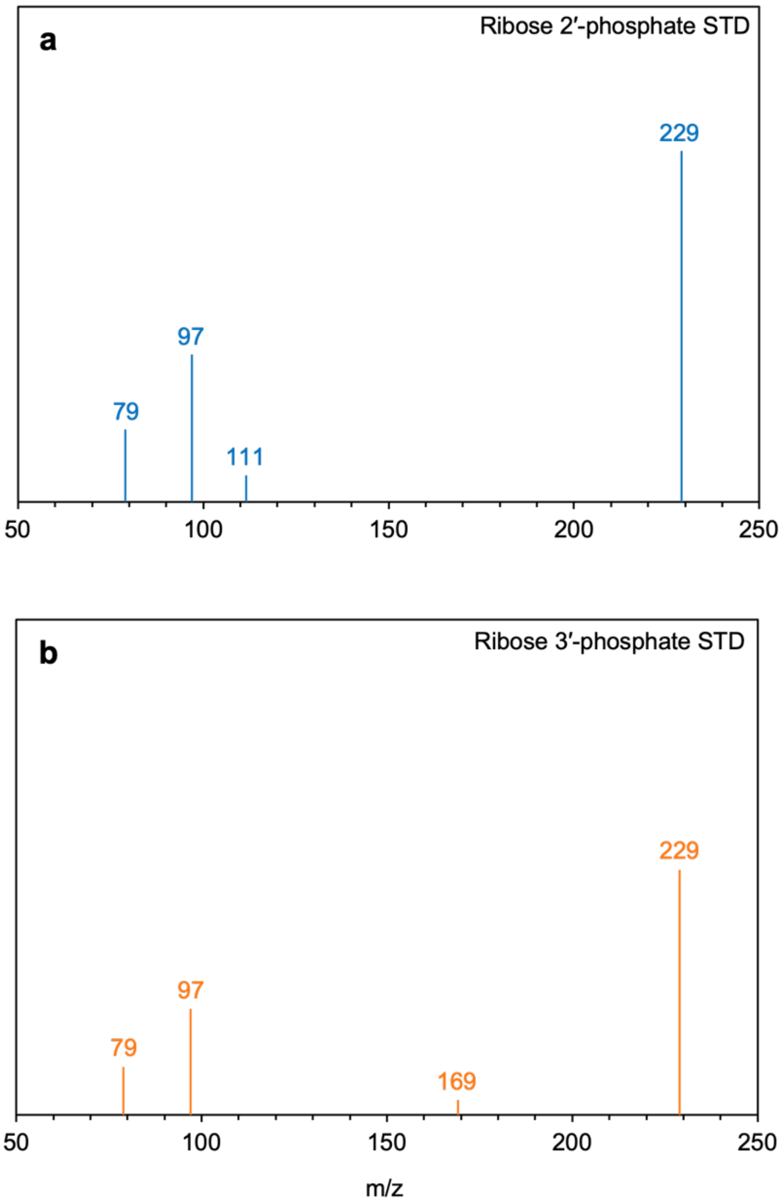

Fig. S1. Fragmentation spectra of ribose-phosphate standards (m/z: 229.1). (**a**) Ribose 2′-phosphate. (**b**) Ribose 3′-phosphate.

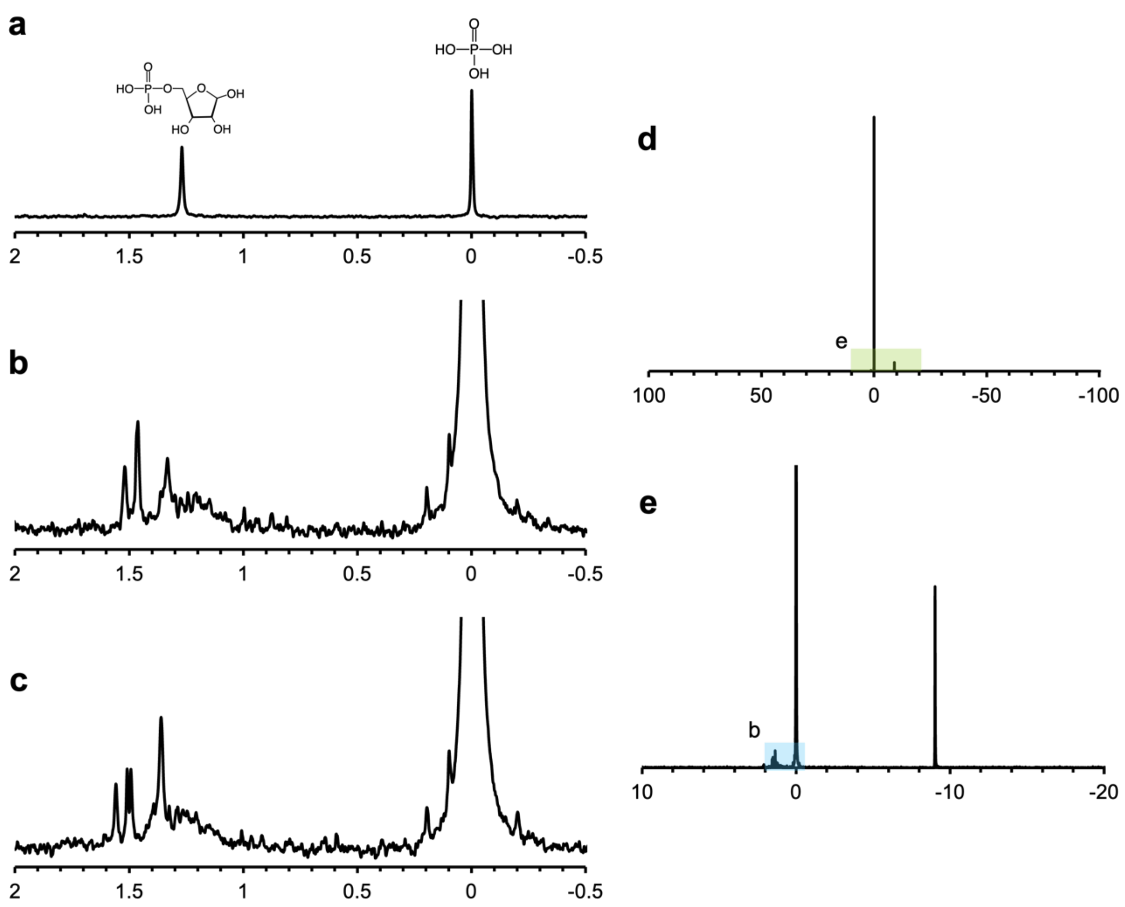

Fig. S2. ^{31}P-NMR spectra of Ribose-5′-phosphate. (**a**) Standard phosphates. (**b**) Reaction product. (**c**) Reaction product added by the standard phosphates. (**d**) Reaction product full spectrum. The green shaded area is magnified in (e). (**e**) Magnified spectrum of (d). The blue shaded area is magnified in (b).

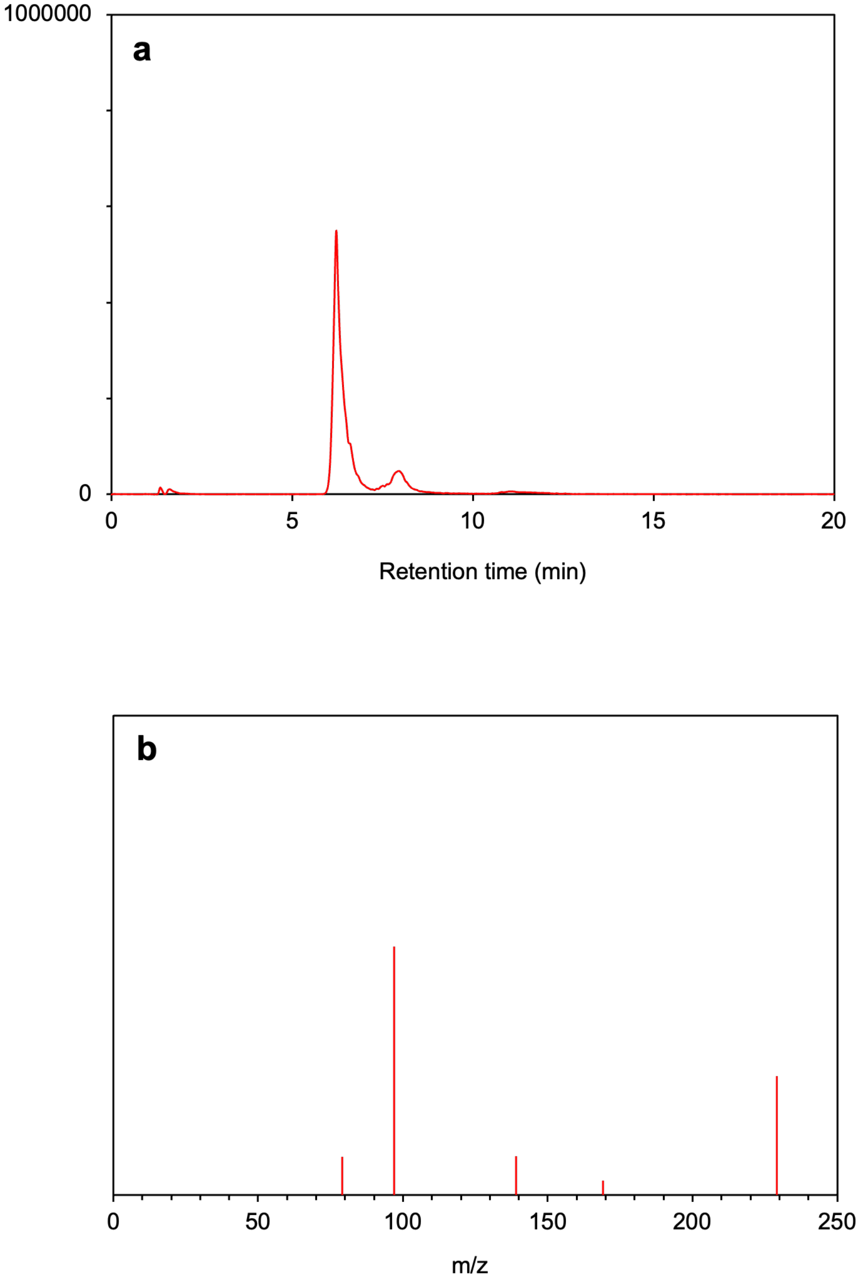

Fig. S3. The full LC-MS chromatogram and the fragmentation spectrum of ribose phosphate in the experimental product. (**a**) LC-MS chromatogram (m/z: 229.1). (**b**) Fragmentation spectrum.

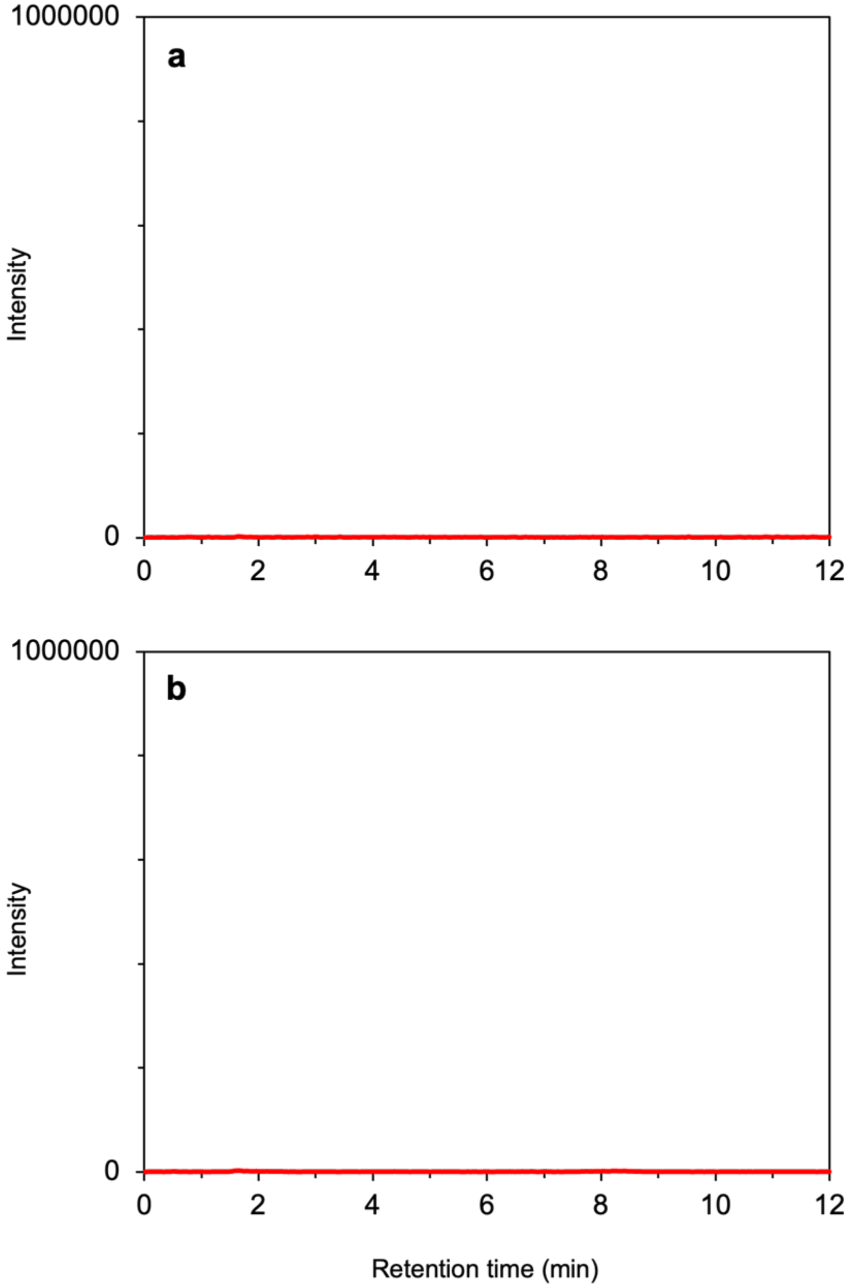

Fig. S4. LC-MS chromatogram that shows the absence of ribose phosphate in the product of the control acidic incubation experiment (*m/z*: 229.1). (**a**) Product of acid incubation. (**b**) Product of acid hydrolysis of the starting materials.

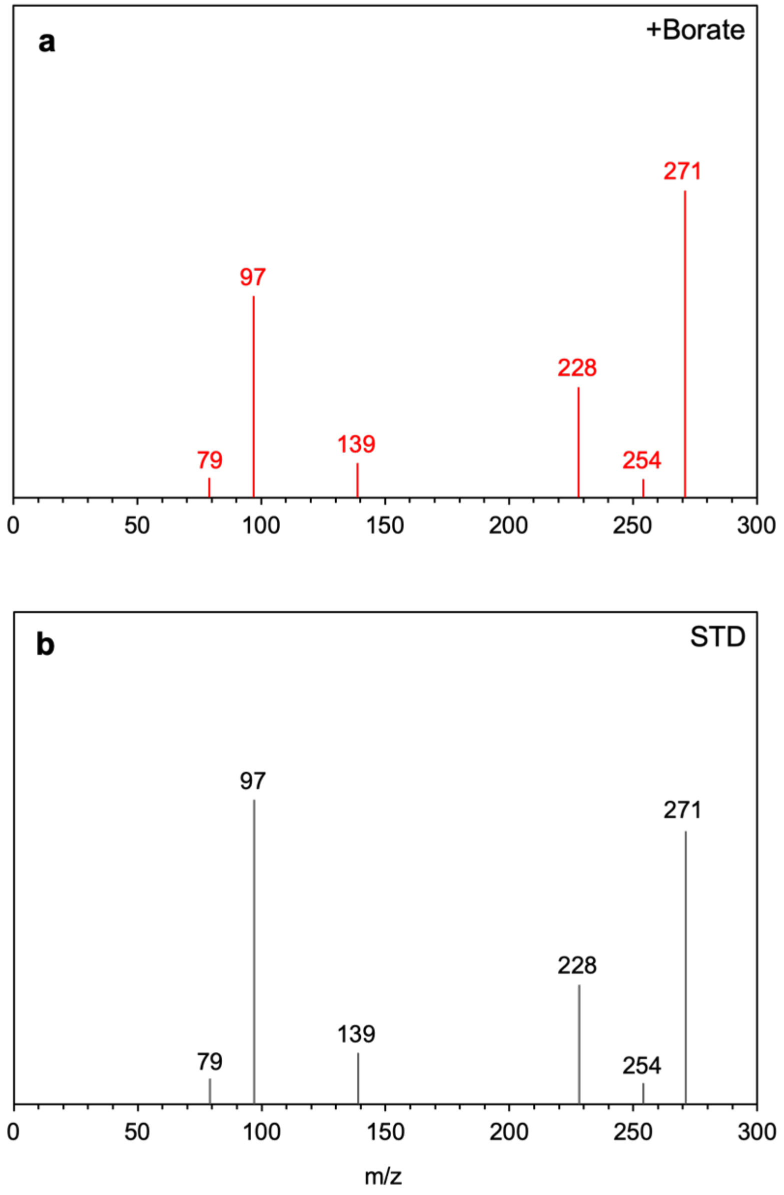

Fig. S5. Fragment mass spectra of phosphorylated ribosylurea (*m/z*: 271.1). (**a**) Product obtained from reaction containing borate. (**b**) Synthetic standard.

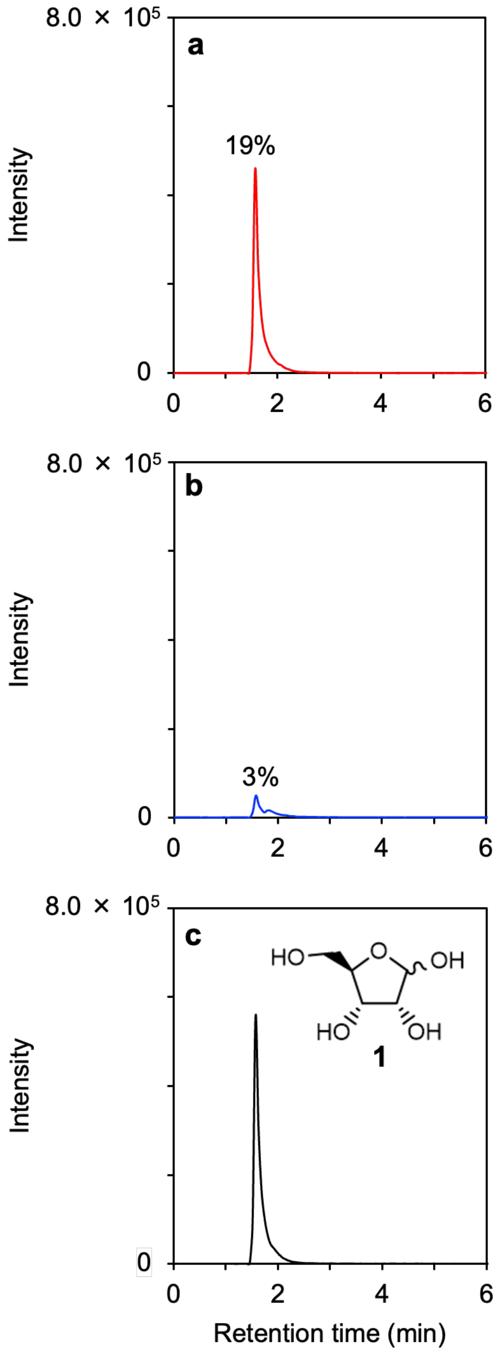

Fig. S6. LC-MS analysis of the residual ribose after acid hydrolysis (*m/z*: 149.1). (**a**) Reaction with boric acid. (**b**) Reaction without boric acid. (**c**) Ribose standard.

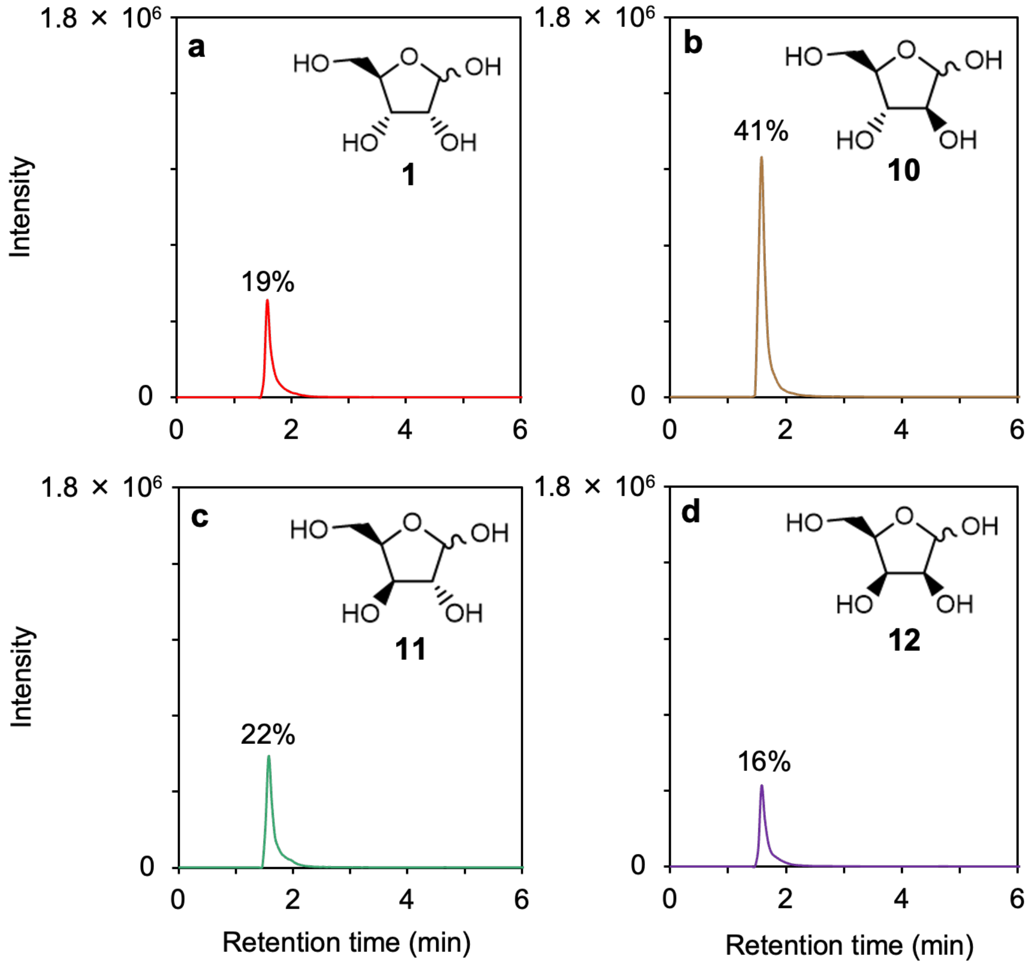

Fig. S7. Residual amounts of pentoses after the reaction with borate (*m/z*: 149.1). (**a**) Residual ribose. (**b**) Residual arabinose. (**c**) Residual xylose. (**d**) Residual lyxose.

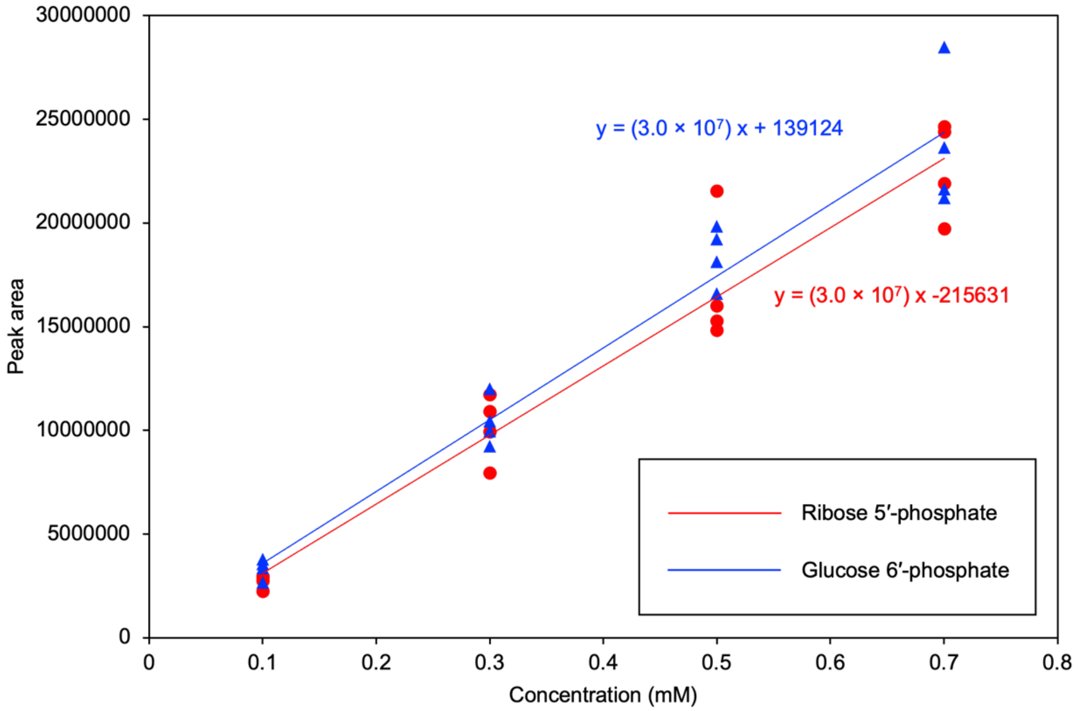

Fig. S8. Calibration curves for ribose 5′-phosphate and glucose 6′-phosphate. Phosphates of arabinose, xylose, and lyxose can be regarded as having almost the same ionization efficiencies as that of ribose 5′-phosphate because ribose 5′-phosphate and glucose 6′-phosphate have almost the same ionization efficiency.

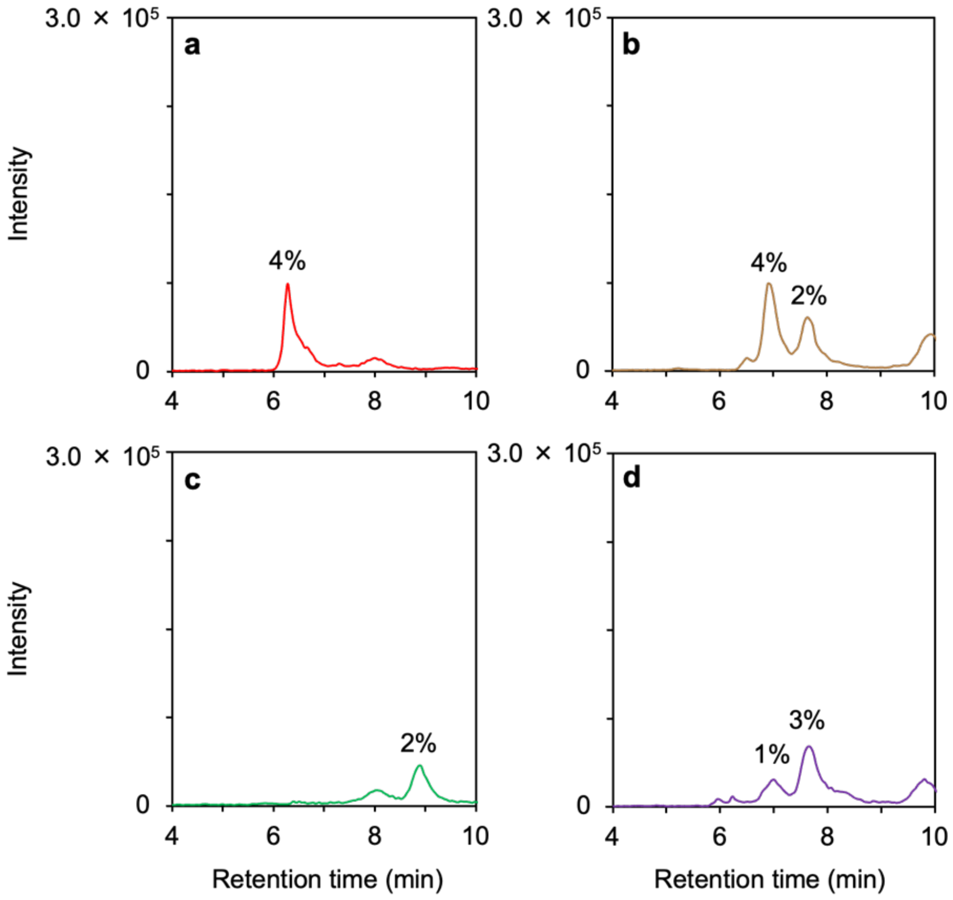

Fig. S9. Yields of pentose-phosphates in the reactions without borate (*m/z*: 229.1). (**a**) Ribose-phosphates. (**b**) Arabinose-phosphates. (**c**) Xylose-phosphates. (**d**) Lyxose-phosphates.

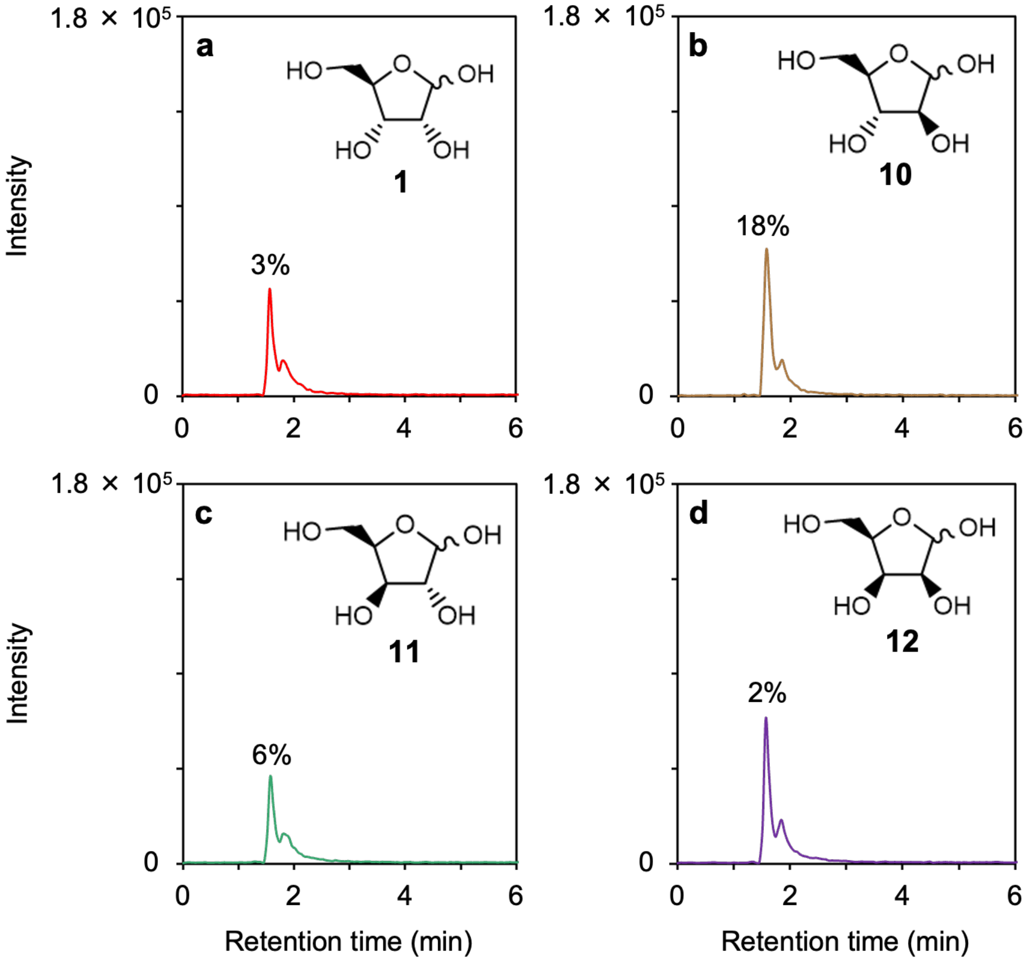

Fig. S10. Residual amounts of pentoses after the reaction without borate (*m/z*: 149.1). (**a**) Residual ribose. (**b**) Residual arabinose. (**c**) Residual xylose. (**d**) Residual lyxose.

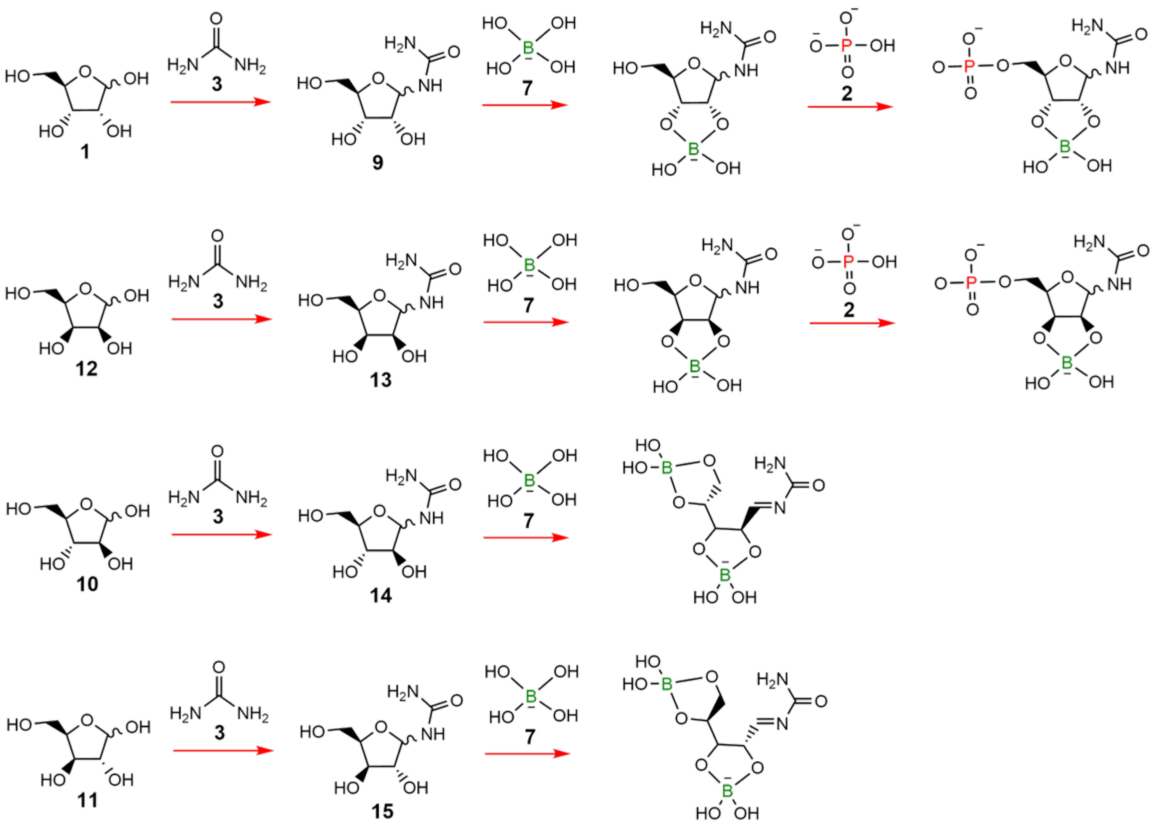

Fig. S11. Possible phosphorylation route for borate-combined ureido pentoses. The structures of ureido pentoses are an example of possible structure.
